# A Mediation Appraisal of Neuropathic-like Symptoms, Pain Catastrophizing, and Central Sensitization-Related Signs in Adults with Knee Osteoarthritis—A Cross-Sectional Study

**DOI:** 10.3390/jpm15010022

**Published:** 2025-01-10

**Authors:** Fausto Salaffi, Marina Carotti, Sonia Farah, Carlo Ciccullo, Antonio Pompilio Gigante, Francesca Bandinelli, Marco Di Carlo

**Affiliations:** 1 Rheumatology Unit, Dipartimento di Scienze Cliniche e Molecolari, Università Politecnica delle Marche, “Carlo Urbani” Hospital, 60035 Jesi, Italy; fausto.salaffi@gmail.com (F.S.); sonia.farah91@gmail.com (S.F.); 2 Dipartimento di Scienze Radiologiche, Ospedali Riuniti di Ancona, Università Politecnica Delle Marche, 60121 Ancona, Italy; marina.carotti@gmail.com; 3 Clinical Ortopaedics, Dipartimento di Scienze Cliniche e Molecolari, Università Politecnica Delle Marche, 60121 Ancona, Italy; carlo.ciccullo@yahoo.it (C.C.); a.p.gigante@staff.univpm.it (A.P.G.); 4 IRCCS INRCA, 60124 Ancona, Italy; 5 Rheumatology Department, USL Tuscany Center, Santa Maria Nuova Hospital, 50143 Florence, Italy; francesca.bandi@gmail.com

**Keywords:** knee osteoarthritis, neuropathic pain, central sensitization, pain catastrophizing

## Abstract

**Objective.** To investigate the relationships among neuropathic pain (NP), pain catastrophizing (PC), and central sensitization (CS) in relation to functional status and radiological damage in patients with knee osteoarthritis (OA). **Methods.** This cross-sectional study included knee OA patients derived from an observational cohort. The Spearman correlation test was used to analyze the relationship between the Western Ontario and McMaster Universities Osteoarthritis Index (WOMAC) and the PainDetect Questionnaire (PDQ), Central Sensitization Inventory (CSI), and Pain Catastrophizing Scale (PCS). The Kruskal–Wallis test was employed to compare WOMAC scores according to CSI categories. A multivariate analysis was conducted to identify predictors of functional ability, with the WOMAC score as the dependent variable and the independent variables including pain-related indices such as PCS, PDQ, and CSI, along with Kellgren–Lawrence (K-L) grading and demographic characteristics. **Results.** This study included 149 patients (76.5% female; mean age 71.5 years; mean duration of pain 8.1 years). In total, 23.5% exhibited NP, 30.9% showed PC, and 33.6% had CS. Higher mean values of WOMAC were correlated with CSI categories (*p* < 0.0001). WOMAC showed a significant relationship with CSI (rho = 0.791; *p* < 0.0001), PDQ (rho = 0.766; *p* < 0.0001), and PCS (rho = 0.536; *p* < 0.0001). In the multiple regression analysis, WOMAC was independently associated with CSI (*p* < 0.0001), PDQ (*p* < 0.0001), and PC (*p* = 0.0001). No association was observed between the K-L grading and the other variables. **Conclusions.** A reduced functional capacity in patients with knee OA is correlated with the presence of NP, PC and CS, without being significantly associated with radiological damage.

## 1. Introduction

Osteoarthritis (OA) is the most prevalent joint condition worldwide [1]. The overall prevalence of knee OA is estimated to be 14.6%, across a wide range of definitions of the disease [2,3,4]. In subjects aged 70 to 74, the prevalence of knee OA rises to up to 40% [5]. Compared to males, females have a higher prevalence of knee OA [6].

Globally, 25% of this population has functional limitations that hinder their ability to perform daily tasks, and 80% experience movement constraints [7,8]. Therefore, it is essential to investigate various aspects of functionality in knee OA patients. Reports of joint discomfort and radiographic findings often differ. When diagnosis is based solely on clinical signs and symptoms, the prevalence in adults is found to be 10% lower [9].

In Nottingham, UK, a survey of people aged 40–79 found that 37.7% of those who reported knee pain had no radiographic evidence of OA, while 58.7% of those who reported no knee pain also had no radiographic evidence [10,11]. In another study, about half of the women aged 45 to 64 with radiographic knee OA reported no pain [12]. Moreover, radiological progression is not necessarily associated with a progression of pain [13].

This observed disparity may be attributed to a modified mechanism of pain perception, as pain sensitization is becoming a crucial factor in determining knee OA-related pain [14]. Altered central pain modulation includes the overactivation of ascending and descending pain facilitatory pathways, as well as compromised function of brain-orchestrated descending anti-nociceptive (inhibitory) mechanisms [15,16].

Overall, nociceptive transmission is enhanced rather than inhibited in OA. A shift in the balance between facilitatory and inhibitory pathways, along with altered brain sensory processing, contributes to this enhanced transmission [16]. These findings suggest that it is incorrect to categorize OA pain as exclusively nociceptive; both local and central nociceptive and neuropathic pathways contribute to OA pain [17]. OA results from a complex biological process involving structural alterations in tissues such as the meniscus, cartilage, bone, ligaments, capsule, synovial membrane, and periarticular muscles [18,19,20]. In rat models of kneeOA, loss of the subchondral bone joint has led to severe damage to the sensory nerve fibers innervating the knee, causing neuropathic pain (NP) [21]. Additionally, prolonged nociceptive input may alter central pain processing in the brain, increasing the risk of developing NP. It is estimated that 34% of patients with knee OA experienced pain that included NP [22]. Patients with knee OA who exhibit a widespread medial posterior pattern are more likely to experience neuropathic-like symptoms than those with medial anterior pain [23]. Patients with NP exhibit multimodal hyperalgesia, increased pain sensitivity, and more marked functional impairment [24,25]. Therefore, assessing the impact of NP on knee OA patients’ functionality can aid in their therapeutic care.

Additionally, central sensitization (CS) signs and symptoms have been observed in patients with knee OA [26]. CS, defined as the central nervous system’s amplified response to peripheral input, is a significant concept in pain medicine. It has enhanced the understanding of pathophysiological aspects of common conditions, such as vulvodynia, headaches, chronic pelvic pain, fibromyalgia, irritable bowel syndrome, and other overlapping conditions collectively known as central sensitivity syndromes (CSS) [27]. Symptoms of CS include hyperactivation of pain-facilitating pathways and compromised operation of the brain’s descending inhibitory systems [28]. The identification of CS in individuals with structural diseases like OA has helped to explain why some patients do not respond well to nonsteroidal anti-inflammatory medications or joint replacement surgery, necessitating therapy focused on CS. Individuals with OA who exhibit altered central pain modulation before surgery are more likely to experience adverse outcomes following total knee arthroplasty (TKA) [29,30]. A screening tool called the Central Sensitization Inventory (CSI) has been developed to determine if patients have CS [31].

Pain modulation and perception are influenced by affective states, such as pain catastrophizing (PC), in OA-related outcomes [32,33]. PC is a pattern of cognitive and affective pain appraisal characterized by a tendency to focus on and magnify the threat value of painful stimuli and to feel powerless in the face of pain [34]. There is strong evidence linking PC with worse functional performance, increased pain, and self-reported disability [35,36,37]. Although PC and CS are connected, limited research has explored potential interactions between the two. PC involves an exaggerated negative orientation towards pain stimuli and experiences, characterized by ruminating on painful sensations, magnifying the threat of pain, and feeling powerless over pain [38]. Pre-operative PC can predict several outcomes, including decreased quality of life (QoL), uncomfortable body sites, poor mood, and the emergence of chronic post-operative pain [38,39,40].

Although the negative influence of these conditions, considered individually, on outcomes in patients with knee OA is well known, little has been studied regarding how the concurrent presence of NP, CS, and PC impacts functional capacity and whether it correlates with radiological damage. Based on these considerations, the aim of this study was to investigate the signs and symptoms of NP, PC, and CS in patients with knee OA and their relationship with radiological damage and performance indicators of health.

## 2. Methods

### 2.1. Data Source and Subjects

The study population comprised participants from the Research on Osteoarthritis Against Frailty (ROAF) study, an observational cohort study of older adults with OA conducted at the Rheumatology Unit of the Università Politecnica delle Marche, “Carlo Urbani” Hospital, Jesi (Ancona), Italy. The primary objective of the study cohort was to identify independent or combined risk factors for OA in a sample of Italian adults with knee OA. To be eligible for enrollment, patients were required to meet the American College of Rheumatology (ACR) classification criteria for knee OA [41]. All participants had experienced knee pain for longer than three months.

The present study is a cross-sectional evaluation of secondary data extracted from the original study. The administration of questionnaires and data collection were conducted by two authors (SF and MDC), who were not involved in the recruitment of patients for the study.

Prior to clinimetric assessment, participants underwent a washout period equivalent to five half-lives of their analgesic or nonsteroidal anti-inflammatory drug (NSAID) medication. They were instructed to refrain from taking these medications for 12 h before testing; however, they were permitted to use paracetamol (acetaminophen) for analgesia if needed during the washout period.

Exclusion criteria included alcohol abuse, psychiatric disorders, planned or previous TKA, concurrent systemic inflammatory rheumatic diseases, coexisting fibromyalgia and any medical comorbidity that would prevent full participation in study procedures (e.g., terminal conditions such as end-stage renal disease, heart failure, or malignancy). All participants provided written informed consent.

### 2.2. Variables

#### 2.2.1. Anthropometric and Demographic Variables

Demographic and anthropometric data were collected through patient interviews. Age and duration of pain were recorded in years. Educational levels were categorized based on the Italian school system: level 1 corresponds to elementary education (0–8 years), level 2 to secondary education (9–16 years), and level 3 to high vocational education/university education (>17 years). Body mass index (BMI) was classified according to World Health Organization (WHO) criteria as normal (18.5 to <25 kg/m^2^), overweight (25 to <30 kg/m^2^), and obese (≥30 kg/m^2^). Weight and height were measured in person at the time of questionnaire administration.

Comorbidities were assessed using the modified Rheumatic Disease Comorbidity Index (mRDCI) [42]. The original Rheumatic Disease Comorbidity Index (RDCI), developed by Michaud and Wolfe, is a self-report tool designed to evaluate the impact of comorbidities on physical disability and health-related QoL (HRQoL) based on the scoring of 11 weighted conditions. The mRDCI has been adapted to include obesity (defined as BMI >30 kg/m^2^) and kidney disease (defined as a glomerular filtration rate <60 mL/min/1.73 m^2^) [43]. The mRDCI is calculated using the following formula: one point for lung disease, myocardial infarction, other cardiovascular diseases or stroke, hypertension, ulcer or other gastrointestinal disease, kidney disease (two points if severe), BMI > 30 kg/m^2^ (two points if BMI > 35 kg/m^2^), and one point for each of the following: diabetes, fracture, depression, and cancer. The mRDCI has been validated and applied in various rheumatological contexts [44,45,46].

#### 2.2.2. Radiographic Assessment

Knee radiographs were obtained using weight-bearing, semiflexed views [47]. A musculoskeletal radiologist (MC), who was blinded to the subjects’ clinical status, assessed images using the Kellgren and Lawrence (K-L) grading system [48]. The X-rays used in this study were typically taken within one year (mean 7.5 ± 3.8 months) prior to the questionnaire assessments, as indicated by previous research [47,49].

### 2.3. Questionnaires

A comprehensive assessment of OA requires consideration of factors that affect functional performance. Consequently, a set of patient-reported questionnaires was administered to explore the study objectives. These questionnaires included the CSI to measure central sensitization of symptoms, the Pain Catastrophizing Scale (PCS) to assess catastrophizing cognitions, and the PainDetect Questionnaire (PDQ) to evaluate pain characteristics. Among the available measures, the PDQ is considered the most suitable for assessing pain in OA [50,51]. Additionally, the Western Ontario and McMaster Universities Osteoarthritis Index (WOMAC) was used to assess pain, stiffness, and physical function in participants with knee OA [52]. Brief descriptions of these questionnaires are provided below.

#### 2.3.1. Central Sensitization Inventory (CSI)

The CSI is a screening tool designed to identify symptoms of CSS and consists of two sections. Part A includes 25 questions related to symptoms of CS, each rated on a 5-point Likert scale ranging from 0 to 4. Higher total scores indicate greater symptoms of CS. A cut-off score of 40 out of 100 suggests the presence of CSS [31]. The severity of CSS is categorized as subclinical (0–29), mild (30–39), moderate (40–49), severe (50–59), or extreme (60–100) [53,54]. Section B assesses the presence of previously diagnosed conditions related to CS, which were not explored in this study. The Italian version of the CSI has demonstrated excellent test-retest reliability, strong discriminative power, and high construct validity [55]. Although there is no documented minimal detectable change for the CSI, two studies have identified it as a “good” responsive treatment outcome measure [56,57,58].

#### 2.3.2. Pain Catastrophizing Scale (PCS)

The PCS is a self-report questionnaire designed to evaluate catastrophic thinking about pain and maladaptive coping strategies. It consists of 13 items rated on 5-point Likert scales, with higher scores indicating greater levels of catastrophizing. A total PCS score of 30 or higher indicates clinically significant catastrophizing [59]. The PCS is widely used in chronic pain registries and in the assessment protocols of pain clinics and rehabilitation facilities across North America and Europe [60]. The psychometric properties of the validated Italian version of the PCS are comparable to those of other language versions [61].

#### 2.3.3. PainDetect Questionnaire (PDQ)

The PDQ consists of nine items: seven related to sensory responses and two related to the temporal and spatial characteristics of pain. The scores for the seven sensory items (ranging from 0 for “never” to 5 for “very strongly”), as well as the scores for temporal (−1 to +1) and spatial aspects (0 or +2), are summed to produce a total score ranging from −1 to 38. A score of less than 12 suggests that the pain is unlikely to be neuropathic, a score greater than 19 indicates a high likelihood ofNP, and a score of 13–18 suggests a possible neuropathic component [50]. The Italian version of the PDQ has demonstrated high test-retest reliability and the ability to distinguish between nociceptive and NP [62].

#### 2.3.4. Western Ontario and McMaster Universities Osteoarthritis Index (WOMAC)

The WOMAC is a multidimensional questionnaire designed to assess pain (5 items), stiffness (2 items), and physical function (17 items) in participants with knee and hip OA [52]. The WOMAC has been translated and validated in several languages, including Italian [63]. The questionnaire was administered through face-to-face interviews, with each item weighted equally and scored on a scale from 0 (none) to 4 (extreme). Each domain can be scored separately, and the total score ranges from 0 to 96, where 0 represents the best possible outcome and 96 the worst.

### 2.4. Statistical Analysis

The sample size was calculated for 144 patients, based on an assumed prevalence of 11% for “likely” NP as indicated by the PDQ [64], with a study power of 90% and an alpha error of 5%.

Normality of the data distribution was assessed using the Kolmogorov–Smirnov test. Since the data were not normally distributed, non-parametric techniques were employed, providing a more conservative estimate of statistical significance, even though parametric techniques could have been appropriate for some ordinal-level data. In addition to means and standard deviations, medians and interquartile ranges were reported where applicable. Categorical variables were presented as proportions and absolute values (percentages).

The Spearman correlation test was used to analyze the relationship between WOMAC scores and PDQ, CSI, and PCS scores. The Kruskal–Wallis test, adjusted for ties, was employed to compare the WOMAC according to CSI categories. Finally, a multivariate analysis was conducted to identify predictors of functional ability, with the WOMAC score as the dependent variable. Independent variables included pain-related indices such as PCS and PDQ, CSI, along with K-L grading and demographic characteristics (age, gender, BMI, education level, comorbidity). A *p* value of less than 0.05 was considered statistically significant. All analyses were performed using MedCalc software (Version 21.2.02 for Windows, Brussels, Belgium).

## 3. Results

### 3.1. Anthropometric, Demographic, and Clinical Data

The study included 149 consecutive patients with knee OA, of whom 76.5% were female, with a mean age of 70.1 years. On average, knee painful symptoms persisted for 8.1 years before diagnosis, with a range of 1 to 19 years. Educational attainment was relatively low, with 41.5% of patients having completed only primary school and 9.8% having completed high school. A significant portion of the patients (55.4%) were housewives, and most (73.5%) were married and living with their families.

A BMI indicative of overweight was observed in 59.7% of the patients. Radiological severity was mostly classified as grade 2 (37.6%) and grade 3 (44.9%) on the K-L scale.

According to PDQ scores, the majority of patients exhibited nociceptive (scores 0–12: “nociceptive”, n = 85, 57.0%) or indeterminate pain (scores 13–18: “indeterminate or possible”, n = 29, 19.5%). Of these, 35 patients (23.5%) were considered to have neuropathic-like symptoms (PDQ scores 19–38).

Anthropometric and demographic data, along with CSI, each dimension covered by the PCS, and WOMAC total scores and subscores, are detailed in Table 1.

**Figure 1:**
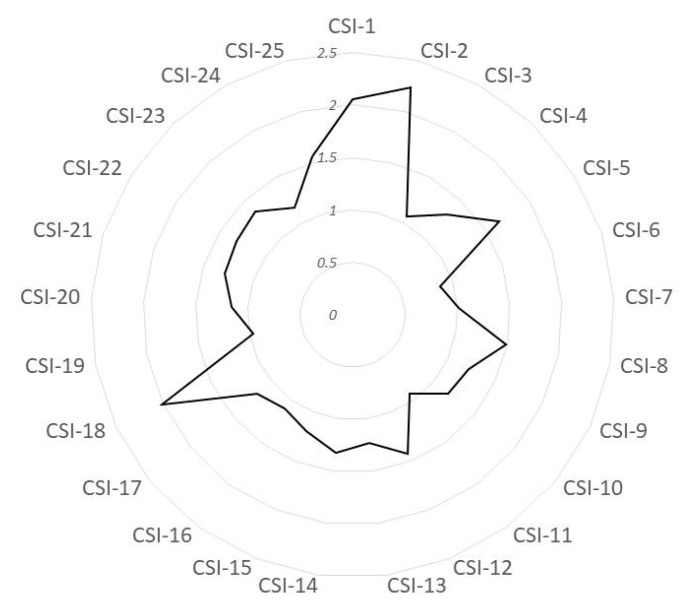
Spydergrams of the Central Sensitization Inventory (CSI) items. The scores are plotted from 0 (best, at the centre) to 10 (worst, at the outside). CSI-1 = I feel tired and unrefreshed when I wake from sleeping; CSI-2 = My muscles feel stiff and achy; CSI-3 = I have anxiety attacks; CSI-4 = I grind or clench my teeth; CSI-5 = I have problems with diarrhea and/or constipation; CSI-6 = I need help in performing my daily activities; CSI-7 = I am sensitive to bright lights; CSI-8 = I get tired very easily when I am physically active; CSI-9 =I feel pain all over my body; CSI-10 = I have headaches; CSI11 = I feel discomfort in my bladder and/or burning when I urinate; CSI-12 = I do not sleep well; CSI-13 = I have difficulty concentrating; CSI-14 = I have skin problems such as dryness, itchiness or rashes; CSI-15 = Stress makes my physical symptoms get worse; CSI-16 = I feel sad or depressed; CSI-17 = I have low energy; CSI-18 = I have muscle tension in my neck and shoulders; CSI-19 = I have pain in my jaw; CSI-20 = Certain smells, such as perfumes, make me filly dizzy and nauseated; CSI-21 = I have to urinate frequently; CSI-22 = My legs feel uncomfortable and restless when I am trying to go to sleep at night; CSI-23 = I have difficulty remembering things; CSI-24 = I suffered trauma as a child; CSI-25 = I have pain in my pelvic area.

Figure 1 displays the subscores for the CSI items. The symptoms with the highest ratings, indicating the greatest impact, were as follows: “I feel tired and unrefreshed when I wake from sleeping” (CSI-1), “My muscles feel stiff and achy” (CSI-2), “I have problems with diarrhea and/or constipation” (CSI-5), “I get tired very easily when I am physically active” (CSI-8), “I do not sleep well” (CSI-12), and “I have muscle tension in my neck and shoulders” (CSI-18).

### 3.2. Correlation Analyses

**Figure 2:**
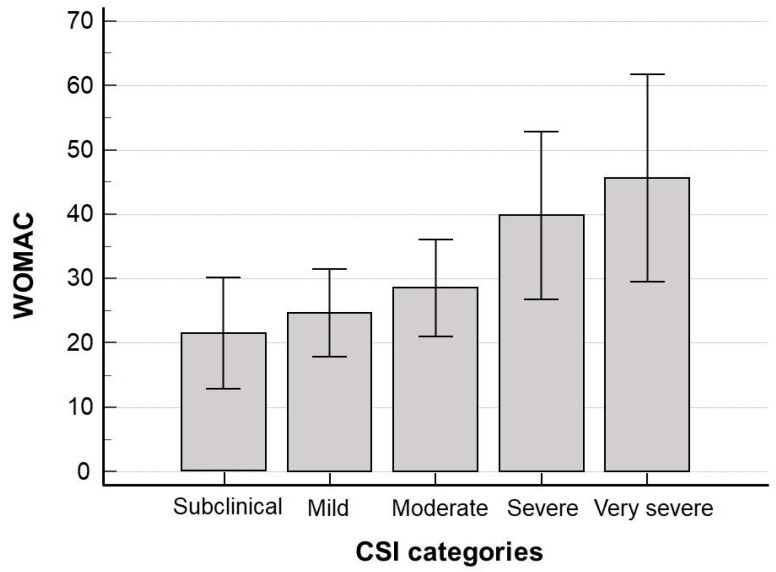
Distribution of the Western Ontario and McMaster Universities Osteoarthritis Index (WOMAC) total scores according to Central Sensitization Inventory (CSI) categories (Ht-values for comparison, Kruskal–Wallis test).

Categorizing patients based on their CSI scores, greater severity of CS was associated with a higher degree of disability (Ht values = 34.871, *p* < 0.0001) (Figure 2).

**Figure 3:**
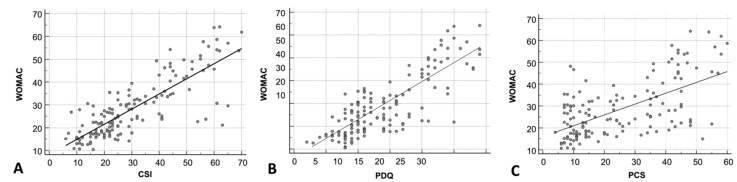
Correlations (Spearman’s coefficients) between Western Ontario and McMaster Universities Osteoarthritis Index (WOMAC) and Central Sensitization Inventory (CSI) (**A**), PainDetect Questionnaire (PDQ) (**B**), and Pain Catastrophizing Scale (PCS) (**C**).

Spearman’s correlation analysis revealed a highly significant association between the WOMAC total score and the CSI score (rho = 0.791, *p* < 0.0001), the PDQ score (rho = 0.766, *p* < 0.0001), and the PCS score (rho = 0.536, *p* < 0.0001) (Table 2) (Figure 3).

### 3.3. Predictors of Impairment of Health Status in Knee-OA (WOMAC Total Score)

Multiple regression analysis revealed that the following variables were independently associated with impairment in knee OA: PDQ (*p* < 0.0001), PCS (*p* < 0.0001), and CSI (*p* = 0.0001) (Table 3).

## 4. Discussion

This study has demonstrated that NP, the presence of CS, and PC are three significant variables that are not only associated with but also serve as predictors of diminished functional ability in patients suffering from knee OA. Given their predictive value, these three variables warrant special attention when it comes to the accurate classification of pain in this patient population. These factors are intricately linked to complex pain mechanisms that extend beyond simple nociception, indicating a multifaceted interplay of underlying processes. To the best of our knowledge, this is the first comprehensive study to evaluate all three of these variables collectively and establish their correlation with reduced functional capacity in patients with knee OA.

Specifically, concerning NP, 23.5% of participants in our research reported a PDQ score greater than 19, suggesting the presence of NP. The prevalence of NP, as indicated by PDQ scores of 19 or higher, varies significantly in patients with knee OA, with reports in the literature ranging from 5% to as high as 67% depending on the study [24,51,65,66]. A recently published systematic review revealed that, according to PDQ, the prevalence of probable NP is 20%, while that of possible NP is 40% [67]. However, this prevalence varies by patient population type: community-based patients exhibited a prevalence of 17% (95% CI: 11% to 24%), hospital-based patients had a prevalence of 25% (95% CI: 15% to 36%), patients involved in randomized controlled trials (RCTs) showed a prevalence of 15% (95% CI: 5% to 26%), and patients with end-stage knee OA had a prevalence of 16% (95% CI: 8% to 26%). Notably, in the general population, these percentages ranged from 1% to 14% [68,69,70].

Moreover, emerging research indicates that following TKA, patients with OA who had preoperative NP tend to report higher levels of postoperative discomfort [71]. Additionally, individuals with NP are more likely to experience multimodal hyperalgesia, heightened pain sensitivity, and more pronounced functional impairment compared to those without NP [24]. This underscores the need for targeted interventions addressing NP in the management of knee OA, as it plays a critical role in patient outcomes.

One of the principal mechanisms underlying the chronification of pain is CS, which may also contribute to the structural brain plasticity associated with chronic pain. The International Association for the Study of Pain defines CS as “an enhanced reactivity of nociceptive neurons in the central nervous system to their normal or sub-threshold afferent input” [72]. Unlike peripheral sensitization, which is characterized by localized pain hypersensitivity confined to the injured area, CS results in remote pain hypersensitivity in areas that are not directly affected and may persist even in the absence of evident peripheral nociceptive activity. This phenomenon highlights the broader and more pervasive impact of CS on the nervous system.

CS is implicated in several chronic pain syndromes, including OA pain, where it has been observed that patients exhibit reduced pain thresholds in regions of the body far from the primary site of clinical pain, which is a hallmark of CS [73]. The underlying pathophysiology of CS involves a complex interplay of glial cells and pro-inflammatory cytokines, which are pivotal in the modulation and amplification of pain signals. Significant advancements in neuroimaging techniques have allowed for the visualization of both white and grey matter alterations in the brain during both induced and resting pain states, further elucidating the structural and functional changes associated with CS. One of the critical features of CS is the impairment of conditioned pain modulation, which is the body’s natural ability to regulate and dampen pain signals [74].

Despite the well-established role of PC as a potent predictor of pain outcomes, including its association with CS, there has been relatively limited research exploring the potential interactions between PC and CS specifically in the context of knee OA. Catastrophizing is recognized as a robust predictor of adverse pain-related outcomes in general [75] and is particularly significant in the context of rheumatoid arthritis pain. This cognitive-affective style is characterized by persistent negative thought patterns, including feelings of helplessness, an exaggerated focus on the severity of pain (amplification), and obsessive rumination about one’s suffering [76]. Furthermore, catastrophizing has been linked to sleep disturbances and indices of CS, suggesting that it may exacerbate the impact of CS on pain perception and overall functional impairment [77,78]. Additional studies confirm that in patients with knee OA and, similarly, in those with chronic low back pain, CS is significantly associated with disability, PC, depression, and anxiety. Depressive symptoms, in particular, are the strongest predictors of elevated scores on the CSI in both knee OA and chronic low back pain patients [79].

Understanding the intricate relationship between CS and PC in knee OA could provide valuable insights into the development of more effective pain management strategies, potentially leading to better outcomes for patients suffering from chronic pain conditions.

TKA is widely recognized as one of the most effective surgical interventions for treating knee OA. However, despite its efficacy, approximately 20% of patients experience unexpected and often distressing levels of discomfort following the procedure. Recent studies have suggested a link between PC and CS and the development of post-TKA discomfort [80]. Notably, research has shown that PC can significantly decrease over time after surgical interventions. For instance, a study found that three years after lumbar spinal stenosis surgery, patients exhibited a marked reduction in PC [81]. Similarly, six months following physical or surgical interventions for persistent anterior knee pain, patients also reported a decrease in PC [82]. These findings indicate that addressing the musculoskeletal issues responsible for pain may also positively influence certain psychological characteristics, suggesting that alleviating the underlying cause of pain can lead to improvements in PC.

The importance of appropriate nosology—accurate classification of diseases and conditions—cannot be overstated in the context of providing effective treatment to patients [83]. Our findings suggest that the inclusion of patients with a centralized pain component may explain the frequently observed discrepancy between radiographic evidence of damage and the reported pain levels in OA research.

This inconsistency between the radiological and clinical manifestations of OA has been corroborated by several other studies [64,84,85]. Neuroimaging studies indicate that in patients with knee OA exhibiting radiological damage severe enough to necessitate TKA, the presence of NP, assessed preoperatively using the PDQ, is associated with altered neural activity in specific brain regions. This includes reduced activity in the rostral anterior cingulate cortex and increased activity in the rostral ventromedial medulla, along with heightened connectivity between these regions, compared to patients with PDQ scores indicative of the absence of NP. Furthermore, preoperative NP pain and increased activity in the rostral ventromedial medulla are correlated with the persistence of moderate-to-severe pain following TKA [84]. These findings underscore the necessity for preoperative stratification in knee OA patients, irrespective of the extent of radiological damage, using tools that evaluate the presence of NP (and CS).

It is becoming increasingly clear that the traditional dichotomy between structural (“organic”) and neurochemical-structural (“functional”) disorders is outdated, as many individuals experience both types of conditions simultaneously [86]. This realization underscores the importance of recognizing that biology inherently includes psychobiology. Consequently, it is important to avoid using terminology that implies patient blame, such as “somatization”, or “somatizer”. Both pharmacological and non-pharmacological approaches—such as person-centered care and the identification of specific subgroups within the patient population—are essential for effective therapy [87].

This study does have limitations. The reliance on self-reported data collection methods may have influenced the reported levels of pain, potentially leading to misclassification. However, any such misclassification would likely attenuate the relationships observed, rather than exaggerate them. Additionally, because the study was cross-sectional, caution is warranted when interpreting the associations found; these associations do not establish causality. Moreover, the patient population in this study, which predominantly consisted of individuals seeking care at a tertiary rheumatology clinic, may not accurately represent the broader population seen in primary care settings. Although patients were evaluated at least 12 h after their last intake of an NSAID, it is also necessary to consider the potential interference of the treatment on the assessment, given that analgesics such as paracetamol were permitted even in close temporal proximity to the evaluation.

## 5. Conclusions

These results suggest that patients with knee OA frequently exhibit enhanced pain processing features that are not exclusively tied to peripheral nociceptive mechanisms. These features—namely, NP, CS, and PC—are predictive of functional impairment without being associated with radiographic damage. As a result, the conventional one-size-fits-all therapeutic approach, which primarily targets nociceptive pain stemming from tissue damage, may not be adequate for this patient population. Identifying and treating knee OA patients with a more individualized approach could potentially improve treatment success rates [86]. The clinimetric tools available to assess NP, CS, and PC have been extensively validated, and their administration requires only a few minutes. Therefore, it is worthwhile to utilize them in patients suffering from chronic pain conditions such as knee OA. Further research is needed to elucidate the physiological mechanisms underlying these associations, with a particular focus on identifying elements of the pain experience that contribute to the heightened risk of disability in individuals with knee OA.

## Figures and Tables

**Table 1:** Demographic, anthropometric, and clinical data of all subjects (149 patients).

Variables	Mean	Median	SD	IQR
Age (years)	71.49	71.00	6.29	67.00–77.00
BMI (kg/m^2^)	27.36	27.43	3.48	25.16–28.99
mRDCI (0–13)	3.10	3.00	1.28	2.00–4.00
duration of pain (years)	8.10	6.00	7.74	2.00–11.25
PDQ	13.73	11.00	6.34	9.00–18.00
unlikely neuropathic pain, n° (%)	85 (57.04%)			
possible neuropathic pain, n° (%)	29 (19.46%)			
likely neuropathic pain, n° (%)	35 (23.48%)			
K-L				
grade 1, n° (%)	27 (18.12%)			
grade 2, n° (%)	56 (37.58%)			
grade 3, n° (%)	66 (44.89%)			
CSI	30.41	25.00	15.98	18.75–41.25
subclinical, n° (%)	61 (40.93%)	18.03	3.07	13.00–25.00
mild, n° (%)	26 (17.44%)	33.65	2.56	32.00–35.00
moderate, n° (%)	35 (23.48%)	41.45	2.61	39.00–43.00
severe, n° (%)	15 (10.06%)	54.86	3.70	51.00–58.00
very severe, n° (%)	12 (8.05%)	63.00	4.57	50.00–64.00
PCS	24.94	21.00	15.83	10.00–39.50
magnification subscore	3.49	3.00	2.49	1.00–5.00
helplessness subscore	12.36	10.00	7.92	5.00–18.00
rumination subscore	8.91	8.00	6.20	3.00–14.00
WOMAC	28.36	24.97	12.98	17.81–34.55
pain subscore	7.79	7.00	4.46	4.00–10.00
stiffness subscore	3.03	2.65	1.76	1.87–3.68
physical subscore	17.53	13.11	10.74	10.09–22.21

Legend. SD = standard deviation; IQR = interquartile range; BMI = body mass index (in Kg/m^2^); mRDCI = modified Rheumatic Disease Comorbidity Index; CSI = Central Sensitization Inventory; PDQ = PainDetect Questionnaire; PCS = Pain Catastrophizing Scale; WOMAC = Western Ontario and McMaster Universities Osteoarthritis Index; K-L = Kellgren and Lawrence.

**Table 2:** Correlations (Spearman’s rho) among functional and pain-related variables.

		CSI	PDQ	PCS
WOMAC	rho *p*	0.791 <0.0001	0.766 <0.0001	0.536 <0.0001
CSI	rho *p*	- -	0.731 <0.0001	0.790 <0.0001
PDQ	rho *p*	- -	- -	0.755 <0.0001

Legend. CSI = Central Sensitization Inventory; PDQ = PainDetect Questionnaire; PCS = Pain Catastrophizing Scale; WOMAC = Western Ontario and McMaster Universities Osteoarthritis Index.

**Table 3:** Multiple regression analysis of the variables predictive of disability, considering WOMAC as the dependent variable.

Independent Variables	Coefficient	Standard Error	t	*p*	r_partial_	r_semipartial_
(Constant)	9.3006					
BMI	0.1265	0.1553	0.815	0.4166	0.0689	0.0327
Gender	−0.3590	1.2688	−0.283	0.7776	−0.0239	0.0113
Age (years)	−0.0707	0.0837	−0.844	0.4000	−0.0714	0.0339
Educational level (years)	0.1045	0.5155	0.203	0.8397	0.0171	0.0081
K-L grade	−0.8687	0.7680	−1.131	0.2600	−0.0955	0.0454
mRDCI	−0.5443	0.4292	−1.268	0.2068	−0.1070	0.0509
CSI	0.4609	0.0580	7.946	<0.0001	0.5589	0.3192
PDQ	1.1703	0.1480	7.908	<0.0001	0.5570	0.3177
PCS	−0.2305	0.0569	−4.051	0.0001	−0.3249	0.1627

Legend. BMI = body mass index (in Kg/m^2^); K-L = Kellgren and Lawrence; mRDCI = modified Rheumatic Disease Comorbidity Index; CSI = Central Sensitization Inventory; PDQ = PainDetect Questionnaire; PCS = Pain Catastrophizing Scale.

## Data Availability

Study data are available upon reasonable request to the corresponding author.
